# Properties of a Three-Component Mineral Road Binder for Deep-Cold Recycling Technology

**DOI:** 10.3390/ma13163585

**Published:** 2020-08-13

**Authors:** Zdzisława Owsiak, Przemysław Czapik, Justyna Zapała-Sławeta

**Affiliations:** Faculty of Civil Engineering and Architecture, Kielce University of Technology, 25-314 Kielce, Poland; owsiak@tu.kielce.pl

**Keywords:** cement bypass dust, cement paste, mortar, mixed mineral binder, physical properties

## Abstract

This study examined the physical properties of a three-component mineral binder that is typically used in deep-cold recycling. Test binders were produced using Portland cement, hydrated lime, and cement bypass dust (CBPD) as a byproduct derived from cement production. The suitability of CBPD for use in road binders was assessed. Effects of the three-component binder composition on the setting time, soundness, consistency, and tensile and compressive strengths of the cement pastes and mortars were determined. The pastes and mortars of the same consistency obtained at different w/b ratios were tested. On this basis, the mixture proportions resulting in road binders satisfying the requirements of PN-EN 13282-2:2015 were determined. By mixing cement, lime, and CBPD during the tests, binder classes N1 to N3 were obtained. The replacement of 40% of cement mass with the CBPD high in free lime produced road binders suitable for recycled base layers. The total content of CBPD and hydrated lime in the road binder should not exceed 50% by mass. The potential risk of mortar strength reduction due to KCl recrystallization was discussed.

## 1. Introduction

Bituminous road surfaces are subject to degradation due to various environmental impacts. Permanent deformation generated in the foundation or mineral base layers is the distress that requires extensive maintenance. One of the techniques recommended for eliminating the cause of permanent deformation is the environmentally friendly deep-cold recycling technology (CR) [1,2,3,4]. Pavement lower layers produced using CR are semi-rigid base courses that are typically made with bituminous binders (such as emulsion or foamed bitumen) and mineral binders (Portland cement, hydrated lime, fly ash, or cementitious dusts) [5,6]. Mineral binders make the base course stiffer, thereby minimizing the possibility of the regeneration of permanent deformation in the layer. On the other hand, an excessive strength of the binder can contribute to over-stiffening of the recycled layer and cause the formation of cracks running through all the layers of the bituminous pavement [2,5]. To counteract this effect and make the mixture more flexible, an appropriate amount of bituminous binder is added [7]. The stiffness of the layer can also be reduced by replacing cement with supplementary cementing materials (SCMs), such as hydrated lime, and with byproducts of the cement manufacturing process, such as cement kiln dust (CKD) and cement bypass dust (CBPD) [8,9,10,11,12,13,14,15].

The use of cement with the addition of byproducts generated during cement manufacture is a relatively recent innovation and requires undertaking further studies. Dusts, such as CKD or CBPD, especially those generated at lower temperatures in modern furnace systems, may have binding properties as they contain clinker phases [8,9]. They may also contain unreacted free lime [9,13]. The presence of these dusts can thus significantly affect the properties of the binder used in the base layer. Reduced reliance on landfill as a dust management option is an additional advantage of this solution [10,13,16]. The quantity of CKD and CBPD generated in the cement clinker manufacturing process is largely dependent on the technology applied. It is usually between 0 and 25% of the clinker mass, as reported by researchers [16,17]. According to the Polish Cement Association, the amount of dust produced during cement manufacturing is decreasing. Their latest report found that the annual quantity of dusts from cement kilns in Poland was about 1200 tons [18]. Compared to 25,000 tons in Oman, 2.7–3.5 million tons in Egypt, 8 million tons in the UK, and 2.5–12 million tons in the US [16,19,20,21], the amount of 1200 tons appears minor. However, as a single cement plant is capable of producing 1000 tons of CBPD daily [22], the reported dust emission rate is not the same as the total dust quantity generated in Polish cement plants and does not include the CKD and CBPD that are recycled back into the kiln system. The amount of dust so used in Poland is much higher and ranges from 9000 to 25,000 tons a year [23]. In 2016, 15,071 tons of CKD and CBPD were reused in the cement production process [24]. However, as recycled dusts lower the cement quality, research is being conducted into new opportunities for dust management [16].

There is a strong body of research exploring alternatives to CBPD utilization [8,11,14,16]. One of important research directions is soil stabilization [13,16,17,25]. According to data reported in 2006, the USA uses more CKD for this purpose than for cement production. The same source mentions the use of dusts in road pavement construction. A number of researchers [21,26,27] have investigated the application of CKD as a filler in asphalt mixtures. Cement bypass dust is not an inert material as it contains phases that have binding properties [9,13,17]. Therefore, it can be used in the production of mineral binders. The high content of chloride and alkalis [9] prevents CBPD from being widely utilized in the production of the classical cement concrete [16]. Other uses for this material are being studied, such as its incorporation in alkali-activated binders [17,19] and in the binders that are not required to have high strength characteristics [17]. The latter mineral binders are chosen for deep-cold recycling [28,29,30].

Proper selection of the proportions of cold recycled mixture components is of key importance in terms of the required base course properties. The semi-rigid base must protect the pavement against permanent deformations, reflective cracking [2,10], and the local reduction in subgrade load support due to groundwater. The required properties of the base layer are achieved primarily by using appropriate proportions of mineral and bituminous binders of known characteristics [2]. The identification of mineral binder properties is problematic when it is composed of different materials [31,32,33,34]. The most popular binder used in CR is Portland cement. Cement increases the stiffness of the recycled base mixture, thereby increasing the risk of reflection crack formation under service loads. Together with the hydrated lime, CBPD is used to reduce the stiffness of the hardened composite [2]. Stiffness reduction with CBPD is associated with strength lowering, as the CBPD provides a weaker skeleton despite its binding properties [9,12,13]. The ability of CBPD to swell while setting is an interesting property [9,11,12] that balances the shrinkage of Portland cement and hydrated lime at the setting stage. By mixing these three binders, setting-related volume changes can be controlled.

This study aimed at investigating the properties of a three-component mineral binder that can be used in cold recycled mixtures. In addition to conventional components, such as Portland cement and hydrated lime, the binder contained CBPD. The three-component binder consisting of cement, hydrated lime, and CBPD has not as yet been studied for use in deep cold recycling. The experimental plan proposed by Atkinson and Donev [36] was adopted to design the composition of three-component mixtures. The plan involved determining an optimum composition of the cement-lime-CBPD binder with respect to its use for cold recycled base course mixtures. Physical and mechanical properties of the pastes and mortars prepared with seven binder blends were determined, as required by PN-EN 13282-2:2015 [37].

This study aimed at investigating the properties of a three-component mineral binder that can be used in cold recycled mixtures. In addition to conventional components, such as Portland cement and hydrated lime, the binder contained CBPD. The three-component binder consisting of cement, hydrated lime, and CBPD has not as yet been studied for use in deep cold recycling. The experimental plan proposed by Atkinson and Donev [36] was adopted to design the composition of three-component mixtures. The plan involved determining an optimum composition of the cement-lime-CBPD binder with respect to its use for cold recycled base course mixtures. Physical and mechanical properties of the pastes and mortars prepared with seven binder blends were determined, as required by PN-EN 13282-2:2015 [37].

## 2. Materials and Methods

### 2.1. Properties of the Components Used in the Binder Preparation

**Figure 1:**
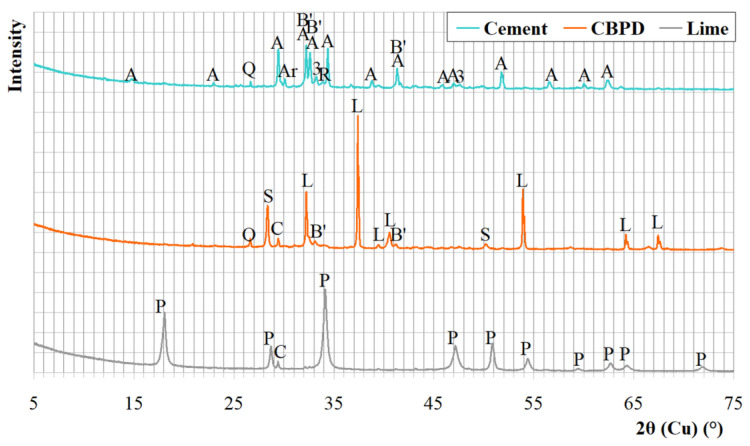
The X-ray patterns of cement CEM I 32.5R, CBPD, and hydrated lime. Designations: A—alite, B’—belite, R—brownmillerite, 3—C_3_A, Ar—arcanite, Q—quartz, G—gypsum, L—free CaO, S—sylvine, P—portlandite, C—calcite.

**Figure 2:**
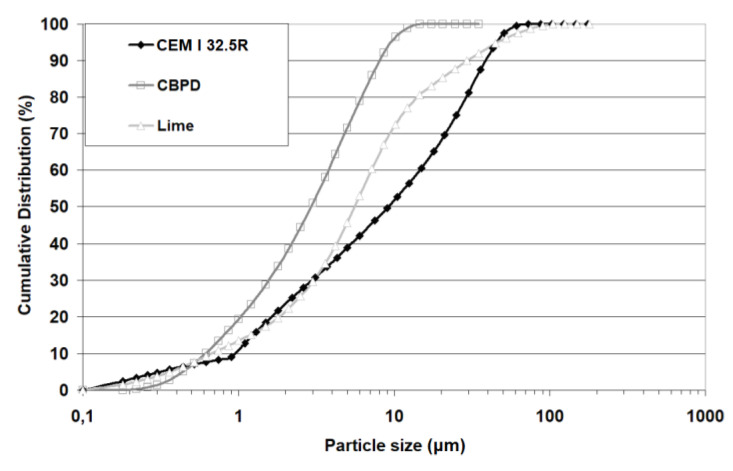
Raw materials’ particle size distributions.

The input materials used for composing the mineral road binders were Portland cement CEM I 32.5R (Cement Ożarów, Ożarów, Poland), hydrated lime (ZSChiM "PIOTROWICE II", Sitkówka, Poland), and CBPD with a high content of free lime (Cement Ożarów, Ożarów, Poland). The chemical composition of the materials is shown in Table 1. The phase compositions determined using X-ray diffraction are presented in the form of X-ray patterns in Figure 1 and in the form of tabulated results of the quantitative analysis in Table 2. The particle size distribution of raw materials is compiled in Figure 2.

Each of the test results of the cement mineralogy revealed a phase composition typical of each respective material. In the CBPD, two phases indicating binding properties were present: CaO and C_2_S, accompanied by sylvine (potassium chloride) and calcite, which were derived from the feed that was calcined before it entered the kiln or were transferred from the kiln by the air stream carrying bridged chlorine compounds. The minor amount of calcite in the hydrated lime may be due to the presence of non-decarbonated raw material or partial carbonation of portlandite. The high content of free lime in the CBPD that was tested is noteworthy when comparing it to other dusts [13].

The particle size distribution of the hydrated lime was found to be similar to that of Portland cement, i.e., from 0 to 100 μm, with lime having more particles in the range of 3 to 45 μm. The inflection above 12 μm on the lime grading curve was probably associated with the formation of lime particle agglomerates. Lime particles larger than 200 μm were excluded from the analysis and were regarded as small particle agglomerates that could reach tens of millimeters. The finest particles, from 0.20 to 18.5 μm, were found in the CBPD. Compared to the cement and lime, the CBPD had more particles in the 0.50 to 18.5 μm range but fewer particles in the 0.20 to 0.50 μm range. All components of the hydraulic binder met the EN 13282-2 [20] standard requirements. The recommended limit for the content of particles more than 90 µm in size is 15%. The cement and the CBPD met the standard requirements with respect to their compositions.

### 2.2. Methodology

#### 2.2.1. Experimental Plan

Fitting the response surfaces to the mixture results is performed in the same way as fitting to the data from the central composite design. However, the mixture data are constrained in that the sum of all the mixture components is always 100%. The three-component mixture can be determined by providing a point in the triangular coordinate system defined by three variables. All experimental designs based on the mixture design require vertex points, that is, mixtures consisting of only one component. In practice, these systems may not be feasible due to cost or other technological constraints. In this experiment, constrained mixture designs were used, i.e., the basic mixture design was modified so that the amount of each component was in the range of 20% to 60%. Ultimately, the research program was subordinated to the constrained mixture design based on the simplex-centroid design [38].

**Figure 3:**
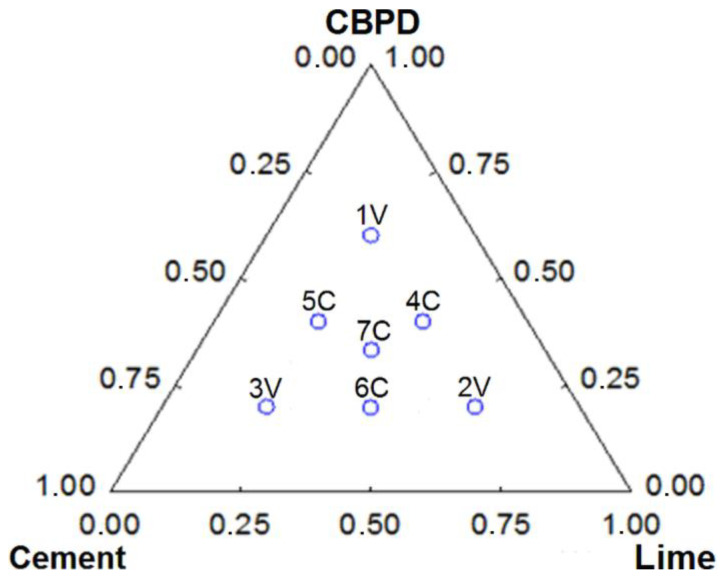
Simplex-centroid design.

The effect of the hydraulic binder composition on its properties was determined by preparing seven different road binders based on the experimental plan. The principles of simplex-centroid design by Atkinson and Donev [36] were adopted to describe the binder composition. The experimental plan assumes the assessment of the effect of the content of individual components and interactions between them on the properties of the binder and allows for evaluating the effect of the binder composition on the specified property at any point within the analyzed region of the experimental plan. The simplex-centroid design is shown in Figure 3. Furthermore, the designations and compositions of the hydraulic binders are shown in Table 3.

Seven road binders were prepared based on the experimental design to comprehensively evaluate the effects of binder components on the properties of hardened mortar. Pure Portland cement was used as the reference binder.

Figure 3 shows the code marking locations for the combination of components included in the universal binder and the method of determining the percentage value of those components. The amount of a given component in the triangle in Figure 3 is the length of the segment, which is the bisector between the neighboring sides of the triangle. The constrained mixture design consisted of estimating pseudo-components and treating the constrained region as the complete design. In practice, an experiment analysis with the use of mixture designs is the multiple (multivariate) regression with the constant component reduced to zero. The effect of the mixture composition on the properties of the innovative binder was assessed based on the analysis of the adequacy of the type of the approximated function of the test object and the estimation of the function coefficients.

The polynomial function was adopted as the approximating function. The degree of the polynomial was dependent on the significance of its form for explaining the variability of the test results. The next stage of the analysis was the estimation of the polynomial coefficients with the degree determined on the basis of the analysis of variance. Parameter approximation was based on the least-squares method (LSM).

#### 2.2.2. Paste and Mortar

The chemical composition of the input materials was analyzed as per PE-EN 196-2 [39]. The phase composition was identified using X-ray diffraction (XRD) on the powder samples. The Empyrean diffractometer (PANalytical, Almelo, Netherlands) was used. The 2θ angle range of 5° to 75° was analyzed with a step size of 0.0167° and a count time of 60 s. The PANalytical XRD analysis software HighScore 4.6 with the International Center for Difration Data (ICDD) database PDF-2 was used for phase identification. Particle-size analysis of the binders was performed using laser diffraction with a Hellos KR laser diffractometer (Sympatec, Clausthal-Zellerfeld, Germany).

The proper amount of mixing water, the initial and final setting times, and changes in the binder volume were determined using the Le Chatelier test (Institute of Ceramics and Building Materials, Cracow, Poland) in accordance with PN-EN 196-3 [40]. The Blaine test (Institute of Ceramics and Building Materials, Cracow, Poland) was used as per PN-EN 196-6 [41] to measure the specific surface area of the binders. These test methods were used to explain the influence of individual constituents on the consistency of the prepared mortars.

The mortar consistency was determined using the penetration test according to PN 85/B-04500 [42] and the flow table test according to PN-EN 1015-3 [43].

These tests were performed to determine the water to binder ratios (w/b) required for the appropriate consistency of the mortars. It is known that the surface area and hence the water demand varies considerably between cement, hydrated lime, and CBPD. Moreover, the presence of a significant amount of free lime found in the composition of the CPBD can contribute to the evaporation of a portion of the mixing water as a result of heat release during hydration [9,12], thereby reducing the effective w/b ratio. There is thus a need to determine the w/b ratio for each binder, which produces pastes and mortars suitable for testing, and in the longer term for use in cold recycling.

Mortar compressive and flexural strengths were measured on 40 × 40 × 160 mm bars at 28 and 56 days according to PN-EN 196-1 [44]. Determining the 56-day strength is essential for checking whether the mixtures meet the requirements for road binders set forth in PN-EN 13282-2:2015 [37]. The 28-day tests are classical strength tests performed for various cement composites and are widely discussed in the literature [44].

## 3. Test Results of Binder Physical and Mechanical Properties

### 3.1. Determination of the Density, Specific Surface, Proper Amount of Water, and Setting Time of Binders

Table 4 presents the results of the tests for density, specific surface, water amount, setting time and binder volume stability.

The density and specific surface of the binders depended on the percentages of the input components. Lime or bypass dusts used as the replacement for cement increased the water demand of the binder. This was related to the considerably lower specific surface and higher density of the cement [17,44]. The presence of CBPD increased the water demand more than the addition of hydrated lime. The initial setting time for all binders was more than 150 min, which is consistent with the requirements of the standard. The presence of hydrated lime in the binder had the strongest effect on the setting-time extension. The shortest setting time was observed for the 3V binder. In addition to cement as the main component, the 3V binder contained small quantities of lime and CBPD. The action of alkalis and free lime present in the CBPD contributed to the faster setting of the binder compared to the Portland cement. At small quantities, the alkalis and free lime can act as cement-setting activators. Likewise, the rise in the sample temperature resulting from the free lime hydration can promote faster setting. As a rule, however, the setting time of the prepared three-component binders was extended. This was due to the fact that the setting times of hydrated lime and CBPD were much longer than that of cement [17,25].

The standard requirement for cement binders is that the change in the Le Chatelier soundness should not exceed 30 mm. Binders 1V and 4C did not meet this condition; all the other binders exhibited swelling within acceptable limits. It was thus evident that a high content of CBPD in binders led to significant changes in their volume. The results obtained for 4C and 5C could be compensated for by adding cement, while the addition of hydrated lime had a negligible effect. The CBDP material was the major contributor to the increase in the binder water demand.

### 3.2. Determining the Mortar Consistency

Mortars containing particular binders having compositions as specified for standard mortars in PN-EN 196-1 were used in the tests for consistency. Table 5 compiles the mean values from three measurements.

Mortars were tested at different w/b values, and the consistency results obtained for mortars with the designed binders were compared with those for the mortar prepared with the cement binder. As in the paste consistency tests, the water demand of the binders increased with a decrease in the cement content. Unlike in the case of pastes, the differences were smaller because the consistency of mortars was largely related to the water demand of the aggregates and the quantity of aggregates was the same in each sample [44]. These results do not confirm the beneficial effect of CBPD on the fluidity of mortars, as found by Sreekrishnavilasam and Santagata [17] for low strength materials. These results confirm the concrete analysis findings presented by Siddique [25], who reported a thicker consistency with the increased content of CKD.

### 3.3. Test Results of Mortar Compressive and Flexural Strengths

**Figure 4:**
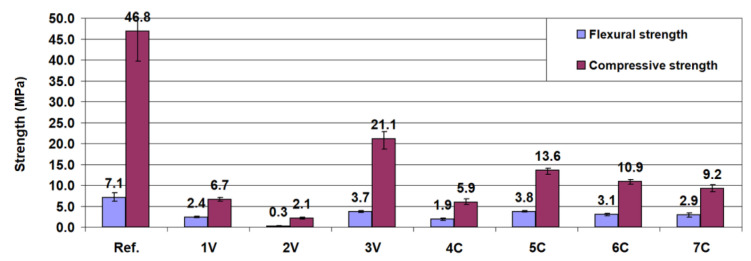
The 28-day compressive and flexural strengths of mortar bars made with the road binder.

**Figure 5:**
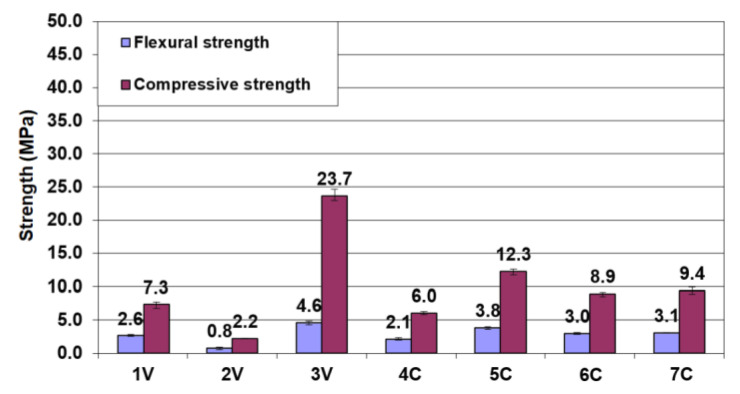
The 56-day compressive and flexural strengths of mortar bars made with the road binder.

Figure 4 and Figure 5 show the results of flexural and compressive strength tests of mortar bars at 28 and 56 days. The results of the strength tests indicate that the use of lime and dust byproducts in the binder led to a significant strength reduction. The mechanical parameters of the reference sample after 28 days were significantly higher than those of other samples. The flexural and compressive strengths of the 3V sample containing 40% less cement were 50% and 55% less, respectively, than the reference sample. Thus, it can be seen that the dependence of strength on the cement content was not linear. The strength of mortars was particularly affected by the addition of hydrated lime to the binder. The presence of CBPD in the binder also reduced the strength of mortars, as confirmed by other studies [14,17,25]. After 56 days, all mixtures except the 2V mixture with the highest proportion of hydrated lime met the standard strength requirements for hydraulic road binders, reaching a minimum strength of 2.5 MPa, thus representing class N1. The highest compressive strength, 23.7 MPa at 56 days, was achieved by the 3V binder, in which Portland cement was the dominant component, representing classes N2 and N3.

In most cases, the comparison between the 28-day and 56-day tests showed only slight strength changes. It can thus be concluded that the binders tested behaved like ordinary Portland cement binders with the strength increase occurring mainly in a period shorter than 28 days. Generally, the changes taking place after this time resulted in an increase in strength, except for binders 5C and 6C. The observed decrease in the strength of these binders may be related to the progressing concrete degradation due to the influence of chlorides from the CBPD [14,17,45].

**Figure 6:**
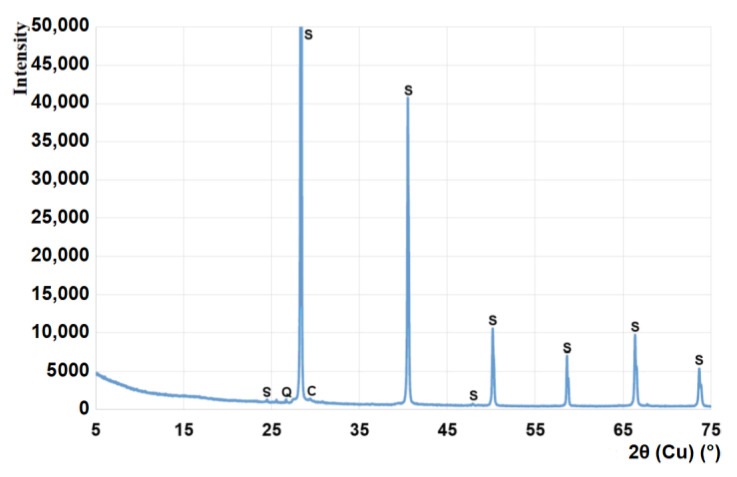
The X-ray pattern of the deposit on the surface of the road binder mortar bars. Q—quartz, S—sylvine, C—calcite.

Within the first 2 days, a white deposit crystallized on the surface of the bars with the high content of cement dust (1V). The X-ray diffraction test results (Figure 6) revealed that the deposit was composed of sylvine crystals. The X-ray pattern also showed single, very weak peaks of other phases in the cement-based mortar, quartz, and calcite.

These findings confirm the recrystallization of sylvine during the setting and hardening of the binder containing CBPD, as found in previous studies [9,12]. In the previous studies, the sylvine recrystallization inside the paste was found to change its microstructure. Small KCl crystals and films formed. As was demonstrated, the crystalline film could also form on the surface of samples, taking the form of large crystals visible to the naked eye. The formation of such large crystals in the limited space of the paste matrix could damage it and thus reduce its strength. This may explain the strength decrease in the period between days 28 and 56 observed for binders 5C and 6C and the lack of dimensional stability of the hardened samples [14,17].

## 4. Discussion

**Figure 7:**
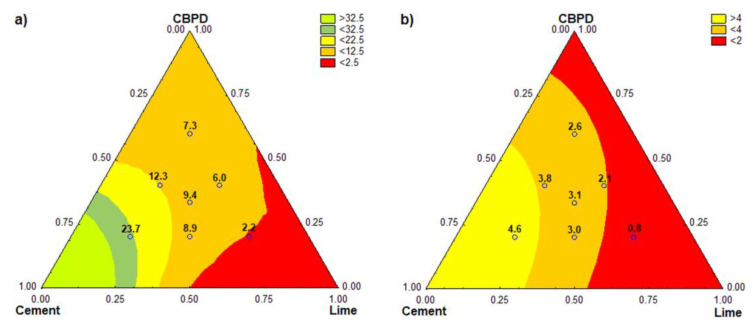
Response surfaces for the variables: (**a**) compressive strength of mortar bars at 56 days and (**b**) flexural strength of mortar bars at 56 days.

A simplex-centroid experimental design was applied to evaluate the results obtained. The second-degree and third-degree models (special cubic) were used to describe the variables. Figure 7 shows the response surfaces of the binder components and their interaction effects on the flexural and compressive strengths of the mortar bars.

Figure 7 shows the road binder composition optimization with respect to strength. Analysis of the effect of the binder composition on its compressive strength (Figure 7a) indicated that cement was primarily responsible for the beneficial properties of the three-component road binder. To meet the requirements of EN 13282-2:2015 for class N1, the road binder must contain up to 60% of hydrated lime at the CBPD content of about 30%, but these proportions were not optimal due to the setting time. Considering the strength parameters, CBPD could replace cement in greater quantities. In theory, all cement could be replaced by CBPD in road binders. This was not possible because of the failure to meet the soundness requirement for binders. For this reason, the CBPD content in the road binder should not be higher than 40% (Table 4). This value, however, is still two times as large as the recommended maximum dosage value for CPBD used as a mineral additive for cement [8]. It is also remarkably larger than the amount (12%) recommended as the requirements for the road binder water resistance [29].

Replacing cement with hydrated lime and CBPD often leads to a significant reduction in binder compressive strength, allowing it to be classified as class N1 only. In order to obtain a higher-class road binder, the minimum required amount of cement should be 50% by mass of the binder.

The flexural strength of concrete was observed to be generally lower than that of the mortar; therefore, the mortar determined the upper limit of the concrete flexural strength [45]. This finding is important when designing pavements on the basis of flexural strength values and plays a role in the case of recycled base course layers. There are no requirements concerning the flexural strength of mortars with a hydraulic road binder.

The results (Figure 7b) show that compared to CBPD, the reduction in flexural strength due to lime was much greater. Theoretically, for the mortar strength to fall below 2 MPa, it is sufficient to replace 55% of the cement CEM 32.5R with hydrated lime. To obtain the same effect with CBPD, the cement replacement quantity should be at least 85%.

## 5. Conclusions

The test results obtained from this study show that:

- An appropriate combination of hydraulic road binder components resulted in the optimal composition for the required mechanical and physical performance in the recycled base course layer.
- An addition of CBPD and hydrated lime to Portland cement allowed for obtaining a mineral road binder class N1 to N3 that met the requirements of EN 13282 2: 2015.
- The presence of CBPD in the mineral binder increased its water demand and volume during setting. It also caused the potassium chloride crystallization that occurred after the binder had set, which was manifested by the formation of a white deposit on the mortar surface.
- The presence of hydrated lime contributed primarily to the extended setting time and reduced the flexural and compressive strength of the mortar.
- The Portland cement content was the primary factor that increased the strength of the cement-lime-CBPD mortar.
- The replacement of Portland cement with 50% hydrated lime and CBPD ensured maintaining the required physical and mechanical properties, as demonstrated by the optimization of the hydraulic road binder composition. For road binder production, the CBPD should not be used in an amount greater than 40% of the binder mass.
- The presence of CBPD reduced the strength of the mortars due to KCl recrystallization.

## Figures and Tables

**Table 1:** Chemical composition of Portland cement CEM I 32.5R and cement bypass dust (CBPD).

Material	Content (%)										
	SiO_2_	Al_2_O_3_	Fe_2_O_3_	CaO	MgO	Na_2_O	K_2_O	Na_2_O_e_	Cl	SO_3_	LOI
CEM I 32.5R	19.70	4.28	2.44	64.50	1.60	0.14	0.79	0.66	0.043	3.33	3.50
CBPD	15.44	3.42	1.77	52.17	1.31	0.26	6.03	4.22	3.53	1.65	14.40

**Table 2:** Phase composition of the road binder components (%).

CEM I 32.5R		CBPD		Lime	
C_3_S (alite)	65.3	Free lime	42.8	Portlandite	97.4
β-C_2_S (belite)	10.0	Sylvine	16.2	Calcite	2.6
C_4_AF	4.4	C_2_S (belite)	33.3		
C_3_A	9.3	Calcite	5.9		
Arcanite	1.3	Quartz	2.7		
Gypsum	1.0				
Calcite	7.7				
Quartz	1.0				

**Table 3:** Designations and compositions of binders (mass%).

Binders	Components		
	Cement	Lime	CBPD
Ref.	1.00	0	0
1V	0.20	0.20	0.60
2V	0.20	0.60	0.20
3V	0.60	0.20	0.20
4C	0.20	0.40	0.40
5C	0.40	0.20	0.40
6C	0.40	0.40	0.20
7C	0.33	0.33	0.33

**Table 4:** Proper amount of water, setting times of individual binders, and the results of the binder volume stability determined in the Le Chatelier ring.

Binder Type	Ref.	1V	2V	3V	4C	5C	6C	7C
Density (kg/dm^3^)	3.05	2.85	2.57	2.89	2.74	2.86	2.75	2.76
Specific area (m^3^/kg)	377	534	576	466	555	501	521	520
w/b	0.27	0.70	0.67	0.45	0.61	0.57	0.50	0.55
Initial setting time (min)	200	265	1050	160	310	265	380	450
Final setting time (min)	265	305	1620	190	630	460	710	800
Setting time (min)	65	40	570	30	320	195	330	350
Soundness (mm)	9	54	22	21	53	17	9	17

**Table 5:** Consistency of mortars under analysis.

Binders	w/b	Flow Table Test	Penetration Test
		(cm)	(cm)
Ref.	0.50	13.5	4.2
1V	0.79	13.5	3.5
2V	0.76	13.5	3.2
3V	0.60	13.5	4.2
4C	0.75	14.0	3.5
5C	0.68	14.0	3.5
6C	0.62	13.5	3.8
7C	0.66	14.0	3.4
